# Louisville Summer School of Medicine

**Published:** 1844-08

**Authors:** 


### LOUISVILLE SUMMER SCHOOL OF MEDICINE.

The lectures in the Louisville Summer School of Medicine will be resumed on the first Monday of September, and be continued until the last of October. Connected with this course, which will consist of three lectures a day, clinical instruction will be delivered at the Marine Hospital. The dissecting rooms will be open the first of October.

